# Objective bayesian analysis for multiple repairable systems

**DOI:** 10.1371/journal.pone.0258581

**Published:** 2021-11-23

**Authors:** Amanda M. E. D’Andrea, Vera L. D. Tomazella, Hassan M. Aljohani, Pedro L. Ramos, Marco P. Almeida, Francisco Louzada, Bruna A. W. Verssani, Amanda B. Gazon, Ahmed Z. Afify

**Affiliations:** 1 Department of Statistics, Federal University of São Carlos, São Carlos, Brazil; 2 Department of Mathematics & Statistics, College of Science, Taif University, Taif, Saudi Arabia; 3 Facultad de Matemáticas, Pontificia Universidad Católica de Chile, Macul, Santiago, Chile; 4 Institute of Mathematics and Computer Sciences, University of São Paulo, São Carlos, Brazil; 5 Department of Exact Sciences, University of São Paulo, Piracicaba, Brazil; 6 Department of Statistics, Mathematics and Insurance, Benha University, Benha, Egypt; Universidad Rey Juan Carlos, SPAIN

## Abstract

This article focus on the analysis of the reliability of multiple identical systems that can have multiple failures over time. A repairable system is defined as a system that can be restored to operating state in the event of a failure. This work under minimal repair, it is assumed that the failure has a power law intensity and the Bayesian approach is used to estimate the unknown parameters. The Bayesian estimators are obtained using two objective priors know as Jeffreys and reference priors. We proved that obtained reference prior is also a matching prior for both parameters, i.e., the credibility intervals have accurate frequentist coverage, while the Jeffreys prior returns unbiased estimates for the parameters. To illustrate the applicability of our Bayesian estimators, a new data set related to the failures of Brazilian sugar cane harvesters is considered.

## 1 Introduction

In view of the opportunity to increase production and reduce costs, with a focus on the new industrial models, the failures that appear in processes and equipment, have to be analyzed, preventive actions must be taken and control must be constant. Failure analysis and prevention makes it a crucial factor within a more productive demand that focuses on reducing operating costs and increasing the quality as well as it is essential that a repair action occurs as soon as possible after each failure. In order to study the analysis of the failures and repair actions, an area of reliability called repairable systems emerged.

In this area, the object of study is the system, which can be machinery, software or electronic equipment, and Ascher and Feingold [1] defined that a system can be considered repairable if its activity is resumed satisfactorily through repair after a failure without the need to replace all the components of the system, that is, repairable systems data are recurrent event data. It is very important to notice the difference between repairable and non-repairable systems because one can draw erroneous conclusions if this difference is not noticed.

Several models emerged with the idea of modeling the effect of the repair action performed on the systems. Aalen [2] was the first author who developed the statistical models based on counting processes for recurrent events. Tomazella [3] described the wide literature of models based on counting processes uses for recurrent event data. Cox and Isham [4] and Cox and Lewis [5] are examples of these models where covariates are present.

The most well-known models are the Minimal Repair (MR), Perfect Repair and Imperfect Repair. The MR is the most analyzed assumption in the literature which describe situations where the repair action is only for the purpose of the system returns to its functionality, so after the repair the system is in the same condition as just before the failure, and this is known in the literature as ABAO—As Bad as Old. This assumption was discussed by [6–10], among others.

The MR is an assumption that the repair maintains the system in the same condition as it was before and it is reasonable for systems that consist of several components and each component has its own failure mode [11]. The failure followed by a repair occurs several times within the study period, and therefore the occurrence of failures has an associated counting process that can be characterized by a Non-Homogeneous Poisson process (NHPP), in which the probability of failure in a short time depends only the system’s age, not on the failure history [12].

The parameter estimates of multiple identical repairable systems have been widely discussed using the maximum likelihood approach and its asymptotic properties, as in Berman and Turner [13], Zhao and Xie [8] and Rigdon and Basu [14]. There are some disadvantages in the frequentist context, since the estimators under the MLEs are biased for small samples and the confidence intervals relies in the asymptotic theory, returning non reliable results. To overcome this problem Bayesian inference can be used, several papers have considered this approach, such as Guida et al. [15] who used several choices of informative and noninformative priors, Sen [16], Yu et al. [17] and de Oliveira et al. [18] that use non-informative prior, and as Kim et al. [19] and Huang [20] that use conjugate prior distribution. dos Reis et. al. [21] used an empirical Bayes approach to modeling power law process for the analysis of repairable systems considering hierarchical model whereas Almeida [22] and Pollo et. al. [23] have considered objective prior to model repairable systems in the context of competing risks. On the other hand, the existing papers do not explore which prior leads to unbiased estimates neither if they have good frequentist performances. It is important to point out that, in many cases the prior distribution may differ according to the choice of the ordination of the parameters, on the other hand, we are interested in estimate all the parameters simultaneously. Objective priors are proposed in the literature as in Jeffreys [24] and Bernardo [25]. The differential of this work is to use these tools with focus on the analysis of multiple repairable systems and also we will use to model the power law process.

In this paper, the main aim is to obtain objective priors for the parameters of the PLP intensities for multiple identical repairable systems. We obtained two different posterior distributions where the posterior obtained with the Jeffreys prior leads to unbiased estimators for the parameters while the overall reference prior returns marginal posterior intervals with accurate frequentist coverage. The obtained posterior distributions are proper and have one-to-one invariance property (for a detailed discussion see Datta and Ghosh [26]. Besides theoretical proofs, a simulation study is conducted to compare the different proposed estimators. Overall, the simulation study confirms that Jeffreys posterior returned unbiased estimators while the reference posterior returned posterior distributions with accurate confidence intervals. The obtained results outperform the ones obtained by classical approach and should be used to compute the estimates of the proposed model.

To exemplify the practical applicability of the proposed modeling, we will present and analyze a new data set consisting of the failure times of sugarcane harvesters machines during a harvest. These machines are subject to failure during their execution and, generally, only the part that broke is replaced when the failure occurs, leaving it in the same stage as before the failure, so minimal repairs are carried out. In addition, during a harvest, each machine is subject to several failures, requiring a model that includes several repairs and therefore we will use modeling for multiple repairable systems.

The paper is outlined in six sections. Section 2 is dedicated to present some useful characteristics and definitions of repairable systems and the minimal repair model. We describe the objective Bayesian theory and the inference of the parameters of the model analyzed assuming objective Bayesian inference in Section 3. We present the simulation study to verify the properties of the founded estimators in Section 4 and we present a real application in Section 5. Some concluding remarks are presented in Section 6.

## 2 Model formulation

### 2.1 Repairable systems

In repairable systems, after the failure occurs, the system is repaired and continues to be observed. Lindqvist [27] states that in modeling the implicit assumption is that the system is repaired and restarted immediately, this means that the repair time is considered so small that it can be discarded. We denote 0 < *T*_1_ < *T*_2_ < … the system failure times measured in global time, that is, accumulated times from the beginning of the system operation.

**Fig 1:**
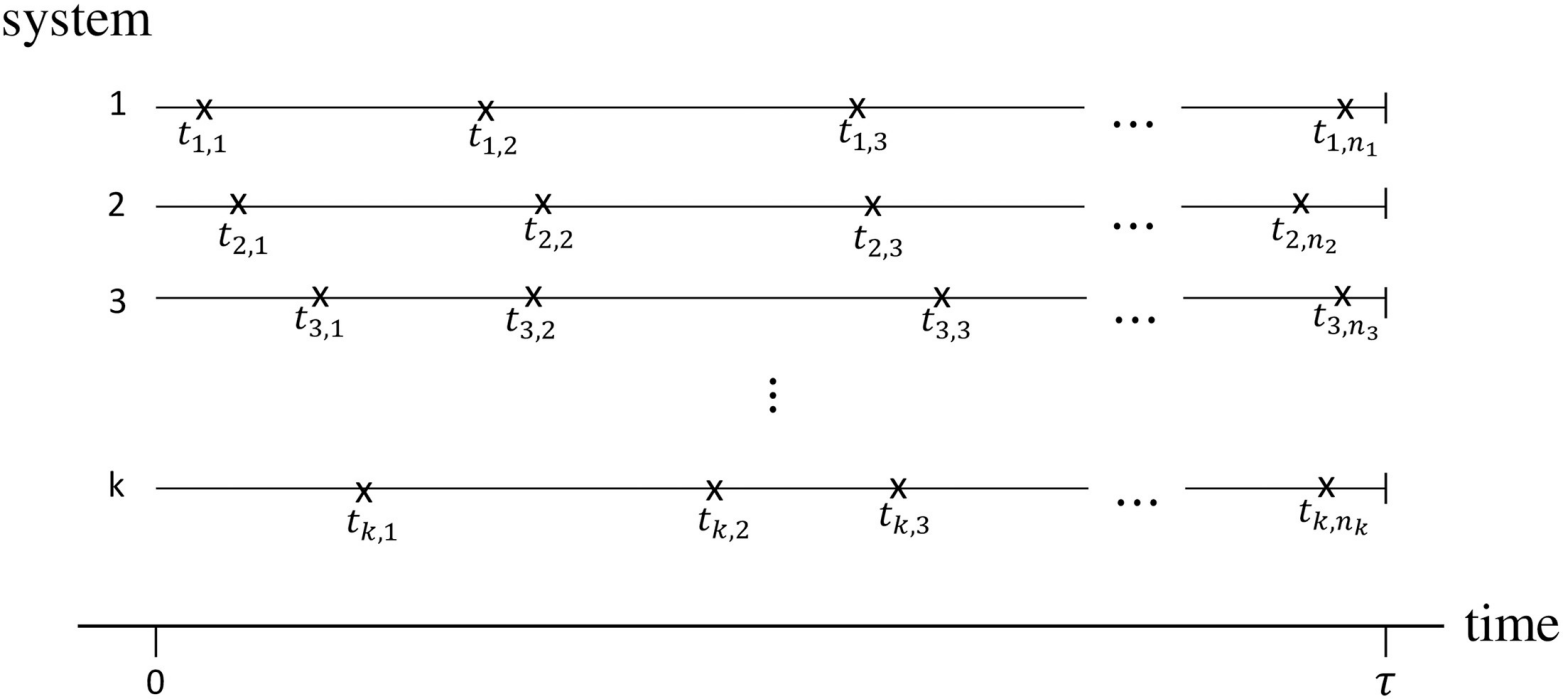
Example of failure time representation (failures are represented by the symbol “×”).

An important concept in repairable systems is truncation. A commonly used truncation is time truncation, where the data set ends at a pre-set time limit *τ*. Let *t*_*i*,*j*_, for *i* = 1, 2, …, *k* and *j* = 1, 2, …, *n*_*i*_, be the observations of the random variable *T* which represents the failure times for the *i*th system, recorded as the time since the beginning of the system $\left(0<t_{i,1}<t_{i,2}<\ldots <t_{i,n_{i}}\right)$. If the *i*th system is truncated by time, it is observed until the predetermined time *τ*, where $0<t_{i,1}<t_{i,2}<\ldots <t_{i,n_{i}}<\tau$, where *τ* is fixed, *n*_*i*_ is random and $t_{i,n_{i}}<\tau$. An example of failure time representation can be found in Fig 1.

The level of repair to be executed depends on the type of failure observed and on the type of system. There are several levels of repairs, from those that correct only the origin of the failure to those that end up improving the system leaving it as good as a new one. Thus, models were constructed that take into account this type of repair. The usual models of the literature are minimal, perfect and imperfect repair.

### 2.2 Minimal repair

The MR focuses on correcting only the cause of the failure, leaving the system in the same condition as it was before. Using the concept of MR, it is possible to simply describe the fact that many real-life repairs bring the system to a condition that is basically the same before failure. This assumption can be used to model a data set where a system component is replaced or repaired. The idea of the MR is just letting the system run as fast as possible and, consequently it is possible to note that the state of the system has not changed.

The NHPP can describe the occurrence of failure, in which the probability of failure in a short period of time does not depend on the previous pattern of failures, but on the system (Muralidharan [12]. Thus, the MR model is a process with intensity function being the actual intensity of NHPP.

One parametric form of NHPP widely used in repairable systems is the power law process. Let *N*(*t*) be the number of failures (NOF) from the beginning of the observation of the system until a time *t*, then, if *N*(*t*) follows a PLP, its function of intensity and the cumulative intensity function [28], are specified by

$$\begin{array}{c} \lambda \left(t\right)=\frac{\beta }{\eta }{\left(\frac{t}{\eta }\right)}^{\beta -1} \end{array}$$ (1)

and

$$\begin{array}{c} \Lambda \left(t\right)={\left(\frac{t}{\eta }\right)}^{\beta },\;\;\beta ,\;\eta \;>0, \end{array}$$ (2)

where *β* and *η* refer to the shape and scale parameters, respectively.

The cumulative distribution function of this model is given by

$$\begin{array}{c} F\left(t\right)=\frac{\Lambda (t)}{\Lambda (\tau )}=\frac{{\left(\frac{t}{\eta }\right)}^{\beta }}{{\left(\frac{\tau }{\eta }\right)}^{\beta }}={\left(\frac{t}{\tau }\right)}^{\beta }, \end{array}$$ (3)

where this function can be used to simulate values of this model.

According to de Oliveira et al. [29] interpreted *η* as the time during which it is expected that there is exactly one failure since E[N(η)]=1, while *β* is a measure that represents the deterioration of the system, so the system is deteriorating when *β* > 1 and the system is improving when *β* < 1.

PLP is usual in the literature of repairable systems according to Crow [28] and its popularity is because its function is flexible according to de Oliveira et al. [29]. PLP can accommodate increasing, decreasing and constant intensities of occurrences, when *β* > 1, *β* < 1 and *β* = 1, respectively.

Rigdon and Basu [14] proposed a reparametrization of the PLP parameters to obtain a better interpretation of the parameters. Since the parameter *η* means the time until the expected NOF is equal to 1, according to de Oliveira et al. [18] this is an interpretation that is difficult to understand. Therefore, the following reparametrization was proposed

$$\begin{array}{c} \delta ={\left(\frac{\tau }{\eta }\right)}^{\beta }=E(N(t))=\Lambda \left(\tau \right). \end{array}$$ (4)

With this reparametrization, the intensity function is given by

$$\begin{array}{c} \lambda \left(t\right)=\frac{\beta t^{\beta -1}\delta }{\tau^{\beta }}, \end{array}$$ (5)

where *β* is a measure of improvement or deterioration of the system, and *δ* is the expected NOF ahead of time *τ*.

#### 2.2.1 The likelihood function of minimal repair

For the construction of the likelihood function (LF), we need to make some assumptions:

- Consider *k* repairable systems, for *k* = 1, 2, …, where the systems are independent and identical, that is, the failure patterns are modeled by the same PLP. This assumption of identical systems is reasonable when all the systems are realizations of the same process, with intensity function;
- The *i*th system is truncated by time, being observed until a predetermined time *τ*, where $0<t_{i,1}<t_{i,2}<\ldots <t_{i,n_{i}}<\tau$;
- *n*_*i*_ failures are observed in the *i*th system, *i* = 1, 2, …, *k*;
- $N=\sum_{i=1}^{k}n_{i}$ is the total NOF observed in the systems;
- Let *t*_*i*,*j*_, *i* = (1, 2, …, *k*) and *j* = (1, 2, …, *n*_*i*_), be observations of the random variable *T* which represents the failure times for the *i*th system, recorded as the times from the beginning of the experiment $\left(t_{i,1}<t_{i,2}<\ldots <t_{i,n_{i}}\right)$;
- Let ***μ*** = (*β*, *η*) be the parameter vector to be estimated.

Just combine the joint probability density of the failure times of the *k* systems to obtain the LF for this process.

Considering time truncated data (*τ*), the NOF (*N*(*t*)) is random, and so it must be considered in the construction of the LF, and it can be written as

$$\begin{array}{c} L\left(\mu |t\right)=\prod_{i=1}^{k}\prod_{j=1}^{n_{i}}[\lambda (t_{i,j})]{\text{e}}^{-\Lambda (\tau )}, \end{array}$$ (6)

where $t=(t_{1,1},....,t_{k,n_{k}})$.

Using the reparametrized power law process (5), the LF for time truncation under the assumption of MR is

$$\begin{array}{c} L\left(\mu |t\right)=\prod_{i=1}^{k}\{\prod_{j=1}^{n_{i}}[\frac{\beta t_{i,j}^{\beta -1}\delta }{\tau^{\beta }}]{\text{e}}^{-\delta }\} \end{array}$$ (7)

and the logarithm of the model’s LF is given by

$$\begin{array}{c} \ell \left(\mu |t\right)=N\;\text{log}\left(\beta \right)+\left(\beta -1\right)\sum_{i=1}^{k}\sum_{j=1}^{n_{i}}\text{log}(t_{i,j})+N\;\text{log}\left(\delta \right)-\beta N\;\text{log}(\tau )-k\delta . \end{array}$$ (8)

Maximum likelihood estimates can be easily found, given by

$$\begin{array}{c} {\hat{\beta }}_{MLE}=\frac{N}{\sum_{i=1}^{k}\sum_{j=1}^{n_{i}}\text{log}(\tau /t_{i,j})}\;\text{and}\;\;{\hat{\delta }}_{MLE}=\frac{N}{k}. \end{array}$$ (9)

## 3 Bayesian objective estimation for model

The statistical methodology depends on the construction of probabilistic models that represent the generating mechanism of any random phenomenon under study to deal with the resulting uncertainty. Nowadays, Bayesian methods are increasingly being used. This methodology usually requires a great computational effort. The computational advances in the last few decades have favoured the use of Bayesian methods, which provide a powerful and flexible alternative to the traditional approaches. Besides their formal interpretation, Bayesian approaches provide parsimonious descriptions of observed data, good statistical properties of the estimates, predictions for missing data, a computational framework for model estimation, forecasts of future data, selection, and validation. In this context, the Bayesian approach has become increasingly easy to be realized and for this reason, new Bayesian techniques have appeared increasingly, and they are widely used.

Under a Bayesian perspective, the inference of a problem basically depends on the posterior distribution of the quantity of interest. This posterior distribution combines the available prior information with the information provided by the data. The elicitation of an appropriate prior is the main task for applied statisticians in practice. This distribution should portray the prior knowledge of the researcher regarding the analyzed subject, however, in practice we not always have some previous knowledge of the subject. Thus, non-informative priors have arisen, which aim to demonstrate the lack of prior knowledge about the parameters. There are several prior techniques that are not informative, but we will focus on the objective prior to having several good properties.

### 3.1 Jeffreys prior

The Jeffreys prior is a non-informative prior proposed by Jeffreys [24], widely used because it is invariant to any one-to-one reparametrization. The Jeffreys prior for a parameter vector ***θ*** is defined in terms of the Fisher information:

$$\begin{array}{c} \pi_{J}\left(\theta \right)\propto {\left|H\left(\theta \right)\right|}^{1/2}. \end{array}$$

To find the Fisher information matrix (FIM), we must find the first and second derivatives of the log-LF (8), given by

$$\begin{array}{c} \frac{\partial \ell (\mu |t)}{\partial \beta }=\frac{N}{\beta }+\sum_{i=1}^{k}\sum_{j=1}^{n_{i}}\text{log}(t_{i,j})-\sum_{i=1}^{k}\sum_{j=1}^{n_{i}}\text{log}(\tau ), \end{array}$$

$$\begin{array}{c} \frac{\partial \ell (\mu |t)}{\partial \delta }=\frac{N}{\delta }-k, \end{array}$$

$$\begin{array}{c} \frac{\partial^{2}\ell \left(\mu |t\right)}{\partial \delta \partial \beta }=\frac{\partial^{2}\ell \left(\mu |t\right)}{\partial \beta \partial \delta }=0, \end{array}$$

$$\begin{array}{c} \frac{\partial^{2}\ell \left(\mu |t\right)}{\partial \beta^{2}}=-\frac{N}{\beta^{2}}, \end{array}$$

and

$$\begin{array}{c} \frac{\partial^{2}\ell \left(\mu |t\right)}{\partial \delta^{2}}=-\frac{N}{\delta^{2}}. \end{array}$$

Then the FIM is

$$\begin{array}{c} H\left(\beta ,\delta \right)=E[\begin{array}{cc} \frac{N}{\beta^{2}} & 0\\ 0 & \frac{N}{\delta^{2}} \end{array}]=k[\begin{array}{cc} \frac{\delta }{\beta^{2}} & 0\\ 0 & \frac{1}{\delta } \end{array}] \end{array}$$

and the Jeffreys prior for *β* and *δ* is

$$\begin{array}{c} \pi_{J}\left(\beta ,\delta \right)\propto \sqrt{f_{1}\left(\beta \right)f_{2}\left(\delta \right)}=\frac{1}{\beta }, \end{array}$$ (10)

where *f*_1_(⋅) and *f*_2_(⋅) are positive functions.

As we know, given the failure times ***t*** and the parameter vector ***μ***, the Bayesian inference procedure is based on the posterior distribution, determined from Bayes’ theorem, which is given by

$$\pi (\mu |t_{i,j})\propto L(\mu |t_{i,j})\pi (\mu ),$$ (11)

where *L*(***μ***|*t*_*i*,*j*_) is the LF and *π*(***μ***) is the prior function.

From (21), using the Jeffreys prior given in (10) and the LF given in (7), thus we can find the posterior distribution, given by

$$\begin{array}{ll} \pi (\beta ,\delta |t) & \propto \prod_{i=1}^{k}\left\{\prod_{j=1}^{n_{i}}\left[\frac{\beta t_{i,j}^{\beta -1}\delta }{\tau^{\beta }}\right]\;\text{exp}(-\delta )\right\}\frac{1}{\beta }\\ & \propto \;\text{exp}\left\{-\beta \left[\sum_{i=1}^{k}\sum_{j=1}^{n_{i}}\text{log}\left(\frac{\tau }{t_{i,j}}\right)\right]\right\}\frac{\beta^{N}}{\beta }\delta^{N}\;\text{exp}(-k\delta ). \end{array}$$ (12)

To identify if the posterior function follows a known distribution function, letting $\beta^{*}=\frac{N}{\sum_{i=1}^{k}\sum_{j=1}^{n_{i}}\text{log}\left(\frac{\tau }{t_{i,j}}\right)}$ be the maximum likelihood estimator of *β*, the posterior distribution is given by

$$\begin{array}{c} \pi \left(\beta ,\delta |t\right)\propto \beta^{N-1}\;\text{exp}(\frac{-N\beta }{\beta^{*}})\delta^{N}\;\text{exp}\left(-k\delta \right), \end{array}$$

and then, we can notice that the marginal posterior distributions are

$$\begin{array}{c} \pi \left(\beta |t\right)\propto \text{Gamma}(\beta |N,\frac{N}{\beta^{*}})\;\;\text{and}\;\;\pi \left(\delta |t\right)\propto \text{Gamma}(\delta |N+1,k), \end{array}$$ (13)

that is, both posterior functions have a gamma distribution, which facilitates the process of finding posterior estimators.

From the marginal distributions (13) we can easily prove that the posterior is proper. Therefore, the Bayesian estimates according to the posterior mode are

$$\begin{array}{c} {\hat{\beta }}_{J}=\frac{N-1}{\sum_{i=1}^{k}\sum_{j=1}^{n_{i}}\text{log}(\frac{\tau }{t_{i,j}})}\;\text{and}\;\;{\hat{\delta }}_{J}=\frac{N}{k}. \end{array}$$ (14)

It is important to point out that

$$\begin{array}{c} E\left[{\hat{\beta }}_{J}\right]=E[\frac{N-1}{N}{\hat{\beta }}_{MLE}]=\beta \;\;\;\text{and}\;\;\;E\left[{\hat{\delta }}_{J}\right]=E[\frac{N}{k}]=\delta . \end{array}$$

### 3.2 Reference prior

The Jeffreys prior might not be adequate in multi-parameter cases, as noticed by Jeffreys’ himself and discussed in details by Bernardo [30, pg. 41] or Kass and Wasserman [31]. To work around this problem, the reference prior was proposed by Bernardo [25], which is a class of objective priors that has minimal influence on the posterior estimates. The main idea is to maximize the expected Kullback-Leibler divergence between the prior and the posterior distribution. In order to find the desirable prior, it is needed to separate the parameters into the parameters of interest and nuisance parameters, according to their order of inferential importance Bernardo [25, 30].

Berger et al. [32] proposed an *overall* prior, that is a unique general reference prior to a multiparametric model, that is, it is a common prior for all parameters of a model and, independently of which parameters of the model were taken to be of interest or of inferential importance, it is unique in the sense of being the same. To this construction, it is particularly studied cases that the FIM is diagonal.

Consider the family *f*(*x*|***θ***) and the parameters vector ***θ*** = (*θ*_1_, …, *θ*_*k*_). Also consider ***θ***_−*i*_ = (*θ*_1_, …, *θ*_*i*−1_, *θ*_*i*+1_, …, *θ*_*k*_).

**Theorem 3.1**. *Berger et al*. [32] *Suppose that the FIM of **θ*** = (*θ*_1_, *θ*_2_, …, *θ*_*k*_) *is of the form*

$$\begin{array}{c} H\left(\theta \right)=\text{diag}(f_{1}\left(\theta_{1}\right)g_{1}\left(\theta_{-1}\right),\ldots ,f_{k}\left(\theta_{k}\right)g_{k}\left(\theta_{-k}\right)), \end{array}$$

*where **θ***_−*i*_ = (*θ*_1_, ⋯, *θ*_*i*−1_, *θ*_*i*+1_, ⋯, *θ*_*k*_), diag *is a diagonal matrix, f*_*i*_(⋅) *refers to a positive function of θ*_*i*_ *and g*_*i*_(⋅) *refers to a positive function of **θ***_−*i*_, *and i* = 1, …, *k*. *Hence, the reference prior, for any ordering of nuisance parameters and any chosen parameter of interest, has the form*

$$\begin{array}{c} \pi^{R}\left(\theta \right)\propto \sqrt{f_{1}\left(\theta_{1}\right)\ldots f_{k}\left(\theta_{k}\right)}\;. \end{array}$$

It can be see that the parameters *β* and *δ* are orthogonal as the FIM is diagonal. Hence, we can use the Theorem 3.1 to find the *overall* reference prior.

From Theorem 3.1, the joint reference prior for *β* and *δ* is

$$\begin{array}{c} \pi_{R}\left(\beta ,\delta \right)\propto \sqrt{f_{1}\left(\beta \right)f_{2}\left(\delta \right)}=\frac{1}{\beta \sqrt{\delta }}. \end{array}$$ (15)

From (21), using the reference prior given in (15) and the LF given in (7), thus we can find the posterior distribution, given by

$$\begin{array}{ll} \pi (\beta ,\delta |t) & \propto \prod_{i=1}^{k}\left\{\prod_{j=1}^{n_{i}}\left[\frac{\beta t_{i,j}^{\beta -1}\delta }{\tau^{\beta }}\right]\;\text{exp}(-\delta )\right\}\frac{1}{\beta \sqrt{\delta }}\\ & \propto \;\;\text{exp}\left\{-\beta \left[\sum_{i=1}^{k}\sum_{j=1}^{n_{i}}\text{log}\left(\frac{\tau }{t_{i,j}}\right)\right]\right\}\frac{\beta^{N}}{\beta }\frac{\delta^{N}\;\text{exp}(-k\delta )}{\sqrt{\delta }}. \end{array}$$ (16)

As we did in Section 3.1, to identify if the posterior function follows a known distribution, letting $\beta^{*}=\frac{N}{\sum_{i=1}^{k}\sum_{j=1}^{n_{i}}\text{log}\left(\frac{\tau }{t_{i,j}}\right)}$, that is the maximum likelihood estimator of *β*, we have

$$\begin{array}{c} \pi \left(\beta ,\delta |t\right)\propto \beta^{N-1}\;\text{exp}(\frac{-N\beta }{\beta^{*}})\delta^{N-1/2}\;\text{exp}\left(-k\delta \right), \end{array}$$

from this it follows that

$$\begin{array}{c} \pi \left(\beta ,\delta |t\right)\propto Gamma(\beta |N,\frac{N}{\beta^{*}})Gamma(\delta |N+\frac{1}{2},k), \end{array}$$

and therefore, the marginal posteriors are

$$\begin{array}{c} \pi \left(\beta |t\right)\propto Gamma(\beta |N,\frac{N}{\beta^{*}})\;\;\text{and}\;\;\pi \left(\delta |t\right)\propto Gamma(\delta |N+\frac{1}{2},k). \end{array}$$ (17)

Similar to the Jeffreys prior based on the marginal distributions (17), we can easily prove that the posterior is proper. Hence, the Bayesian estimates according to the posterior mode are

$$\begin{array}{c} {\hat{\beta }}_{R}=\frac{N-1}{\sum_{i=1}^{k}\sum_{j=1}^{n_{i}}\text{log}(\frac{\tau }{t_{i,j}})}\;and\;{\hat{\delta }}_{R}=\frac{N-1/2}{k}. \end{array}$$ (18)

#### 3.2.1 Matching priors

Frequentist methods, in general, find confidence intervals through asymptotic theory, which often does not guarantee a probability of coverage equal to the desired one for small and moderate samples sizes. To circumvent this problem, formal rules were presented to derive Bayesian interval estimators in order to guarantee the probability of coverage error with *O*(*n*^−1^) in the frequentist sense, that is, for the parameters *θ*_1_ and *θ*_2_, let $\theta_{1}^{1-\alpha }(\pi ,\text{t})|(\theta_{1},\theta_{2})$ be the (1 − *α*)th quantile of the posterior distribution of *θ*_1_,

$$\begin{array}{c} P[\theta_{1}\leq \theta_{1}^{1-\alpha }\left(\pi ,t\right)|\left(\theta_{1},\theta_{2}\right)]=1-\alpha -O\left(n^{-1}\right). \end{array}$$ (19)

The class of priors non-informative *π*(*θ*_1_, *θ*_2_) where the credible interval for a parameter of interest *θ*_1_ has a coverage error in the frequentist sense is known as matching priors, see more in Datta and Mukerjee [33]. According to Tibshirani [34], to find these priors it is necessary to find a parametrization of the model in order to have orthogonal parameters (*υ*, *φ*), on what *υ* is the parameter of interest and *φ* is the nuisance parameter.

Hence, the matching priors have the form

$$\begin{array}{c} \pi \left(\upsilon ,\varphi \right)=g\left(\varphi \right)\sqrt{I_{\upsilon \upsilon }\left(\upsilon ,\varphi \right)}, \end{array}$$ (20)

where *g*(*φ*) > 0 is an arbitrary function and *I*_*υυ*_(*υ*, *φ*) is the *υ* diagonal entry of the Fisher information matrix. To obtain priors with a vector of nuisance parameters follows the same idea.

The parametrization admitted in this paper is orthogonal, which allows us to find matching priors.

**Proposition 3.2**. *The Jeffreys prior* (10) *is a matching prior for β*.

*Proof*. Let *υ* = *β* be the parameter of interest and denote by ***φ*** = *δ* the nuisance parameter. Then, $\sqrt{I_{\upsilon \upsilon }(\upsilon ,\varphi )}=\frac{1}{\beta }$ and *g*(*φ*) = 1, therefore, the Jeffreys prior can be written as (20) and the proof is completed.

**Proposition 3.3**. *The overall reference prior* (15) *is a matching prior for all the parameters*.

*Proof*. If *β* is the parameter of interest and *φ* = *δ*, then the proof is analogous to that for the Jeffreys’ prior when considering $g(\varphi )=\frac{1}{\sqrt{\delta }}$. If *δ* is the parameter of interest and *φ* = *β* is the nuisance parameter. Then, $\sqrt{I_{\delta \delta }(\delta ,\beta )}=\frac{1}{\sqrt{\delta }}$ and $g(\beta )=\frac{1}{\beta }$. Then, the overall reference prior can be expressed as (20) and the proof is completed.

There is another interesting point to note about the choice of the prior distribution. As shown in Llorente et. al. [35], an important quantity for model selection purpose is the Bayesian evidence, also known as marginal likelihood, which is given by the following expression

$$p(\text{t})=\int_{\Theta }L(\theta |\text{t})\pi (\theta )d\theta ,$$ (21)

where ***θ*** ∈ Θ The Bayesian evidence is important to model selection and hypothesis testing because it suffers the dependence on the priors choice (much more than the posterior).

In this respect, the choice of the prior distribution is an crucial issue, because even with strong data, the Bayesian evidence is highly sensitivity to the choice of prior density than the posterior. In Llorente et. al. [35], Spiegelhalter and Smith [36] and Piironen [37], there are interesting discussions of strategies about the choice of the prior, such as the likelihood-based priors, the so-called partial Bayes factors (which uses tempered likelihood-based priors) and the posterior predictive approach. In this sense, the robust priors that are used in this work are also good for model selection and hypothesis testing.

## 4 Simulation study

We conducted a simulation study to compare the different approaches discussed previously. The purpose of this simulation study is to examine numerically the properties of the proposed estimators. More specifically, we will assess the impact of the sample size on the properties of the estimators through metrics such as bias, mean square error and coverage probability.

In this study, the different scenarios were chosen to assess the following aspects: small and large sample sizes (sample size, in multiple repairable systems context, is the number of systems), increasing and decreasing intensity function, more and fewer failures in each system.

The comparison between methods was performed using the bias and the mean square error (MSE) that are computed by

$$\begin{array}{c} \text{Bias}\left(\beta \right)=\sum_{i=1}^{M}\frac{{\hat{\beta }}_{i}-\beta }{M}\;\text{and}\;\text{MSE}(\beta )=\sum_{i=1}^{M}\frac{{\left({\hat{\beta }}_{i}-\beta \right)}^{2}}{M} \end{array}$$

$$\begin{array}{c} \text{Bias}\left(\delta \right)=\sum_{i=1}^{M}\frac{{\hat{\delta }}_{i}-\delta }{M}\;\text{and}\;\text{MSE}(\delta )=\sum_{i=1}^{M}\frac{{\left({\hat{\delta }}_{i}-\delta \right)}^{2}}{M}, \end{array}$$

where *M* is the number of replicates considered and ${\hat{\beta }}_{i}$ and ${\hat{\delta }}_{i}$ are the estimators given in (9), (14) and (18) for MLE, Jeffreys and reference priors, respectively.

Based on these metrics, it is expected that the best estimation method returns both bias and MSE closer to zero. The coverage probabilities (CP) of the parameters are also computed, hence, assuming 95% confidence level, the confidence/credibility intervals should include the true value with the proportion of 0.95. Therefore, the CP is a measure to evaluate the quality of the interval estimates. For the maximum likelihood interval, we considered the asymptotic variances obtained from the FIM to construct the intervals. From the Bayesian approach, we obtained the interval values directly from the quantile function of the gamma distribution.

We consider two scenarios for the different parameters:

- **Scenario 1**: we assume a decreasing intensity function, that is, systems in process of improvement and a situation with more failures, adopting *β* = 0.5 and *δ* = 10;
- **Scenario 2**: we assume an increasing intensity function, that is, the systems are deteriorating and a situation with fewer failures, adopting *β* = 3 and *δ* = 5.

For each scenario, we consider different sample sizes: *k* = 2, 5, 10 and 20 systems. In all cases, we assume that the systems are observed in a fixed period of time, that is, they are truncated at time *τ* = 50. Moreover, in each scenario, *M* = 50,000 replicates were generated.

Considering that the failures follow a NHPP, based on Rigdon and Basu [14], we used the following data simulation algorithm:

**Step 1**: We set the parameter values;
**Step 2**: For each system, we generate the NOF *n_i_* ~ *Poisson*(*δ*), *i* = 1, …, k;
**Step 3**: In the ith system, the failure times $t_{i1},t_{i2},\ldots ,t_{in_{i}}$ were generated through the inverse of the intensity function, that is, $t_{ij}=\tau U_{ij}^{1/\beta }$, where *U_ij_* are random numbers from the Uniform(0,1) distribution;
**Step 4**: We repeat steps 2 and 3 *M* times.

The software R [38] was used to compute the results. The MLEs are compared with the Bayesian estimates that are computed in closed-form expressions, therefore, we did not have to consider the use of Markov Chain Monte Carlo methods.

**Fig 2:**
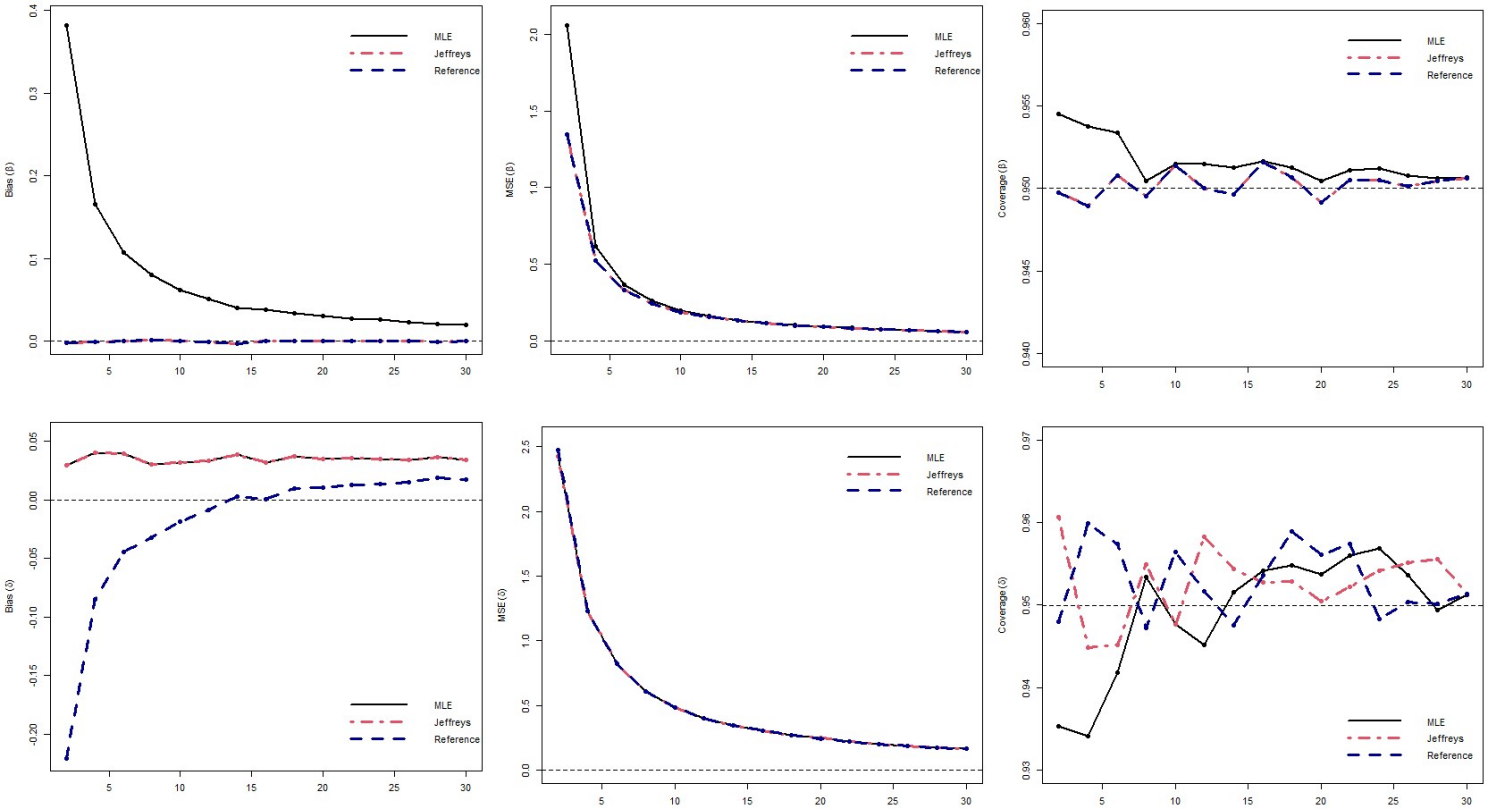
Bias, MSE and coverage probabilities from the estimates with *N* = 50, 000 simulated samples and different estimation methods, considering the sample sizes of *k* = 2, 4, 6, …, 30 for the Scenario 1 (*β* = 3, *δ* = 5).

**Fig 3:**
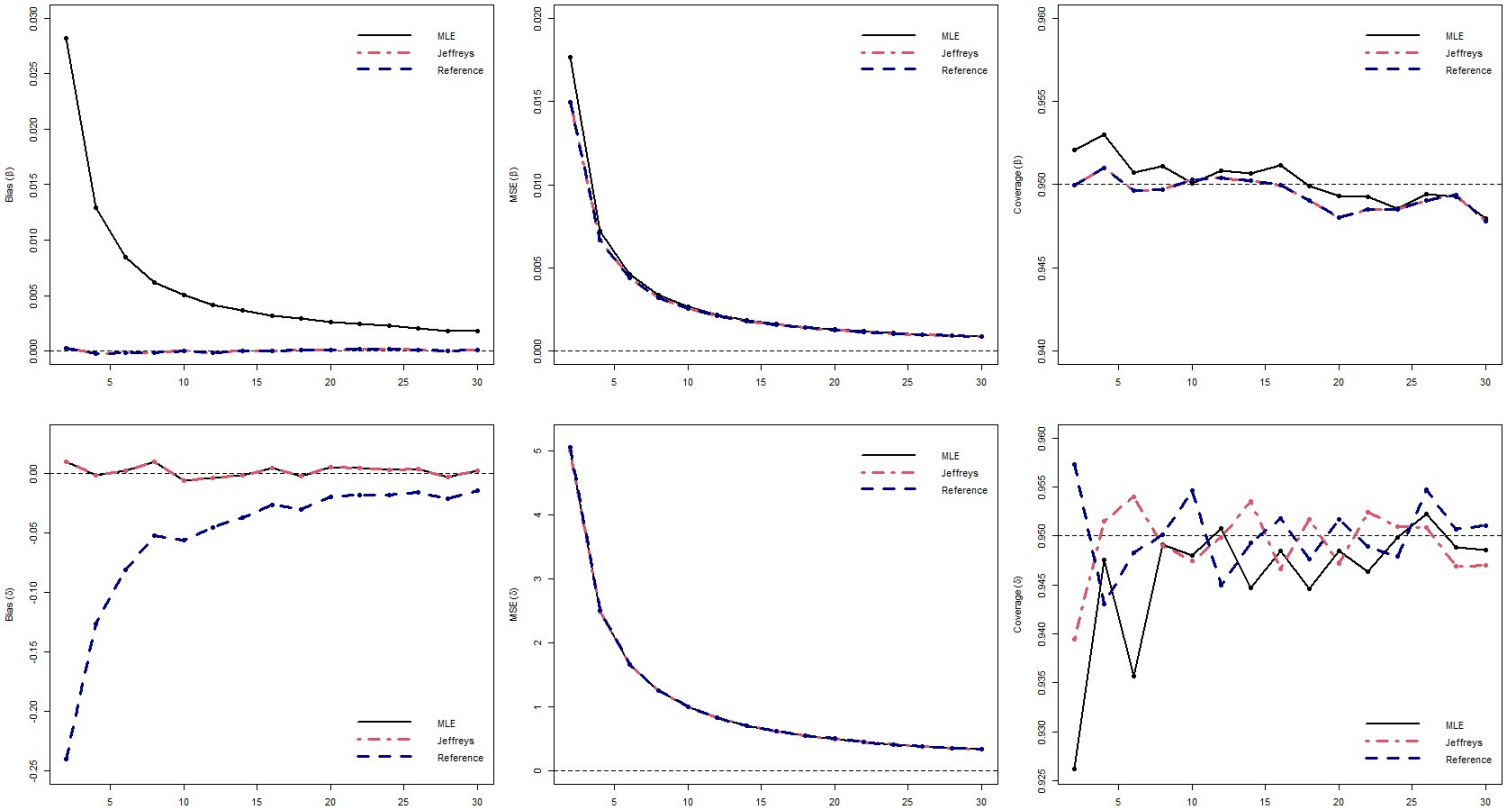
Bias, MSE and coverage probabilities from the estimates with *N* = 50, 000 simulated samples and several estimation approaches, considering the sample sizes of *k* = 2, 4, 6, …, 30, for the Scenario 2 (*β* = 0.5, *δ* = 10).

Table 1 shows the bias and the MSE and Table 2 presents the coverage probability for the two scenarios considering the following sample sizes, *k* = 2, 5, 10, 20 for the three different estimation methods. The Figs 2 and 3 illustrate the bias, MSE and CP for scenarios 1 and 2, respectively.

**Table 1:** The Bias and MSE from the estimates considering the Scenarios 1 (*β* = 0.5, *δ* = 10) and 2 (*β* = 3, *δ* = 5), for different sample sizes *k* = 2, 5, 10 and 20, with *M* = 50,000 simulated samples and several estimation methods.

			k = 2		k = 5		k = 10		k = 20	
Sc.	Par.	Method	Bias	MSE	Bias	MSE	Bias	MSE	Bias	MSE
1	*β*	MLE	0.3825	2.0550	0.1275	0.4603	0.0618	0.2001	0.0307	0.0944
		Jeffreys	-0.0019	1.3454	-0.0020	0.4054	-0.0002	0.1882	0.0003	0.0916
		Reference	-0.0019	1.3454	-0.0020	0.4054	-0.0002	0.1882	0.0003	0.0916
	*δ*	MLE	0.0296	2.4255	0.0386	0.9696	0.0318	0.4834	0.0352	0.2466
		Jeffreys	0.0296	2.4255	0.0386	0.9696	0.0318	0.4834	0.0352	0.2466
		Reference	-0.2204	2.4732	-0.0614	0.9719	-0.0182	0.4827	0.0102	0.2454
2	*β*	MLE	0.0282	0.0177	0.0102	0.0056	0.0051	0.0026	0.0026	0.0013
		Jeffreys	0.0002	0.0149	-0.0002	0.0053	0.0000	0.0026	0.0001	0.0013
		Reference	0.0002	0.0149	-0.0002	0.0053	0.0000	0.0026	0.0001	0.0013
	*δ*	MLE	0.0098	5.0054	0.0021	1.9975	-0.0054	0.9996	0.0055	0.5014
		Jeffreys	0.0098	5.0054	0.0021	1.9975	-0.0054	0.9996	0.0055	0.5014
		Reference	-0.2402	5.0630	-0.0979	2.0071	-0.0554	1.0027	-0.0195	0.5018

Sc. means Scenario; Par. means Parameter

**Table 2:** Coverage probabilities from the estimates considering the Scenarios 1 (*β* = 0.5, *δ* = 10) and 2 (*β* = 3, *δ* = 5), for different sample sizes *k* = 2, 5, 10 and 20, with *M* = 50,000 simulated samples and several estimation methods.

Scenario	Parameter	Method	*k* = 2	*k* = 5	*k* = 10	*k* = 20
1	*β*	MLE	0.9545	0.9510	0.9515	0.9505
		Jeffreys	0.9497	0.9501	0.9514	0.9491
		Reference	0.9497	0.9501	0.9514	0.9491
	*δ*	MLE	0.9353	0.9382	0.9477	0.9538
		Jeffreys	0.9606	0.9461	0.9477	0.9505
		Reference	0.9480	0.9599	0.9564	0.9561
2	*β*	MLE	0.9521	0.9508	0.9501	0.9493
		Jeffreys	0.9500	0.9492	0.9503	0.9480
		Reference	0.9500	0.9492	0.9503	0.9480
	*δ*	MLE	0.9262	0.9397	0.9480	0.9485
		Jeffreys	0.9394	0.9416	0.9475	0.9472
		Reference	0.9574	0.9521	0.9547	0.9517

Analyzing Table 1 we can see that, in relation to *β*, for scenario 1, the MLE has the largest bias and the largest MSE in all sample sizes, while the bias and MSE of the Bayesian estimators are already small with *k* = 2. A similar situation occurs in scenario 2, but in this case, the bias and especially the MSE in all methods are lower than in the previous scenario. When analyzing the parameter *δ*, in both scenarios we see that the estimator using reference posterior presents the greatest bias, however with MSE similar to the other methods and, as the sample size increases, the bias of the estimator using reference posterior gets closer to the other methods. According to Table 2, we can observe that MLE is the method that needs a larger sample size to reach the CP very close to the nominal. We can also note that the Bayesian estimator using reference posterior is the estimator that reaches the CP close to 0.95 with smaller sample sizes. The Bayesian estimator using Jeffreys posterior behaves well in general in all aspects. These results are also illustrated in Figs 2 and 3.

In a general way, it can be seen from the results in Tables 1 and 2 and Figs 2 and 3 that both Bias and MSE are smaller under the Jeffreys posterior when compared with the MLEs. In fact, the results obtained using the Jeffreys posterior are very close to zero, these findings are consistent with the theoretical results that proved that both estimators for *β* and *δ* are unbiased estimators. On the other hand, considering the CPs the reference posterior returned better results when compared with the other estimators, which also confirms our theoretical results. Overall, the Bayesian estimators with the Jeffreys prior returned improved estimates for both parameters as well as good credibility intervals and should be used to obtain the estimates of the parameters. Note that the obtained estimates using the Jeffreys prior returned unbiased estimates for the parameters. Firth [39] showed that if the model belongs to the exponential family, using the Jeffreys prior the obtained the maximum a posterior estimator will be the same as the bias-corrected maximum likelihood estimator discussed by Cox and Snell [40]. In our case, the marginal posterior distributions for the parameters using the Jeffreys prior can be written as gamma distributions, which belong to the exponential family. Therefore, the proposed estimators are the same as if we obtain the Bias corrected MLEs, which confirms the results observed from the simulation study. Note that, the Bayesian approach returned unbiased estimates with accurate credibility intervals even for small samples as we do not need to resort to asymptotic theory.

## 5 Application

In this application section, we analyze the data set collected in Brazilian sugarcane mills related to the breaking of the Chopper blade of sugarcane harvester machines that are used in the production of sugar and alcohol in the states of São Paulo and Parana during the 2014/2015 harvest.

**Fig 4:**
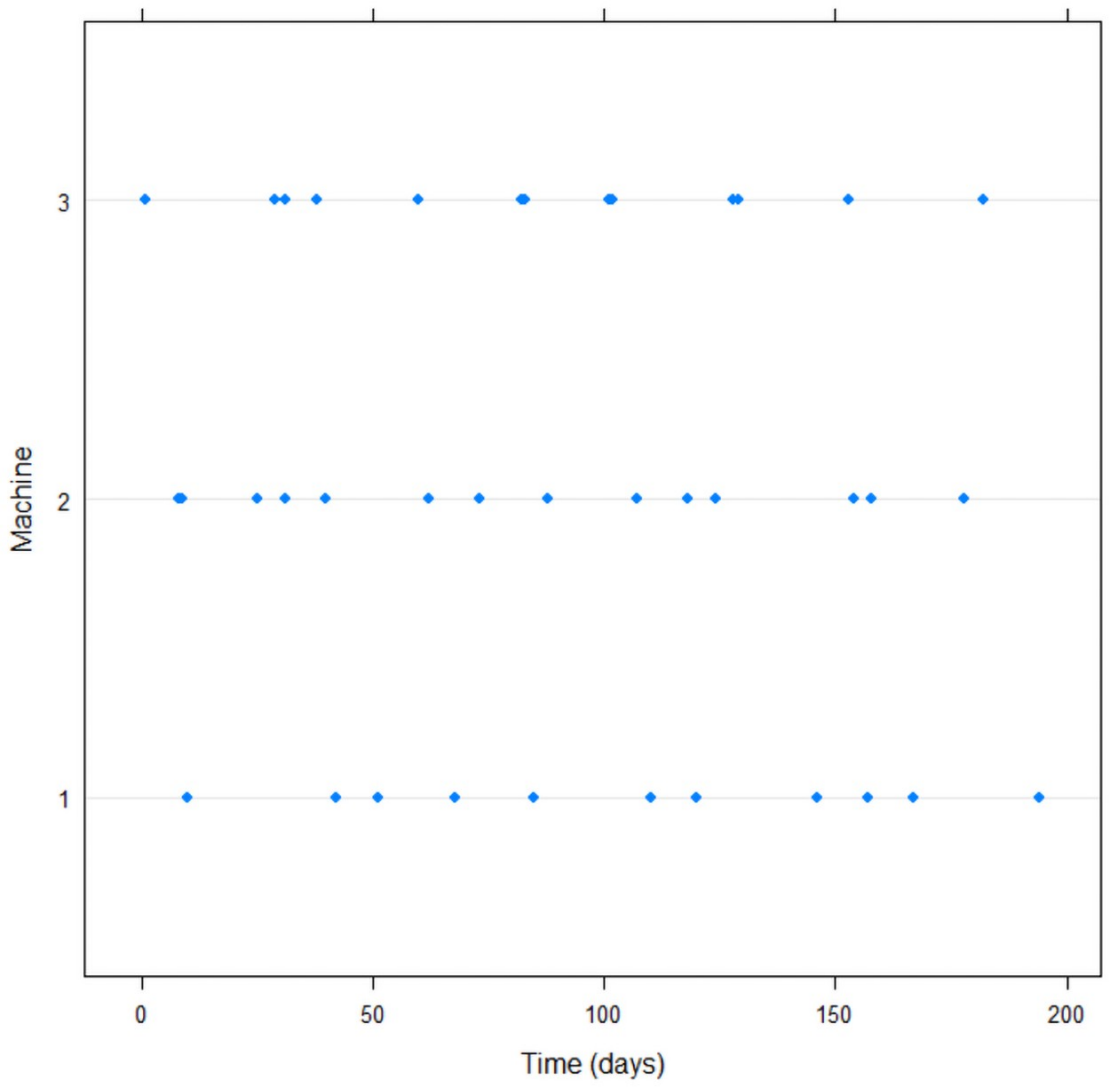
Failure times (in days) for each sugarcane harvester.

This data set describes the failure times of 3 sugarcane harvesters with a total of 38 failures in the Chopper blade, each followed by a repair. Each sugarcane harvester has a different number of repairs, ranging from 11 to 14 failures by harvester. The study was time truncated at 195 days. An outline of the data is presented in Table 3 and its graphical representation is presented in Fig 4.

**Table 3:** Chopper blade break data set (failure times in days).

Harv.	Failures and truncated times (days)														
1	10	42	51	68	85	110	120	146	157	167	194	(195)			
2	8	9	25	31	40	62	73	88	107	118	124	154	158	178	(195)
3	1	29	31	38	60	82	83	101	102	128	129	153	182	(195)	

Truncated times are enclosed between parentheses

**Fig 5:**
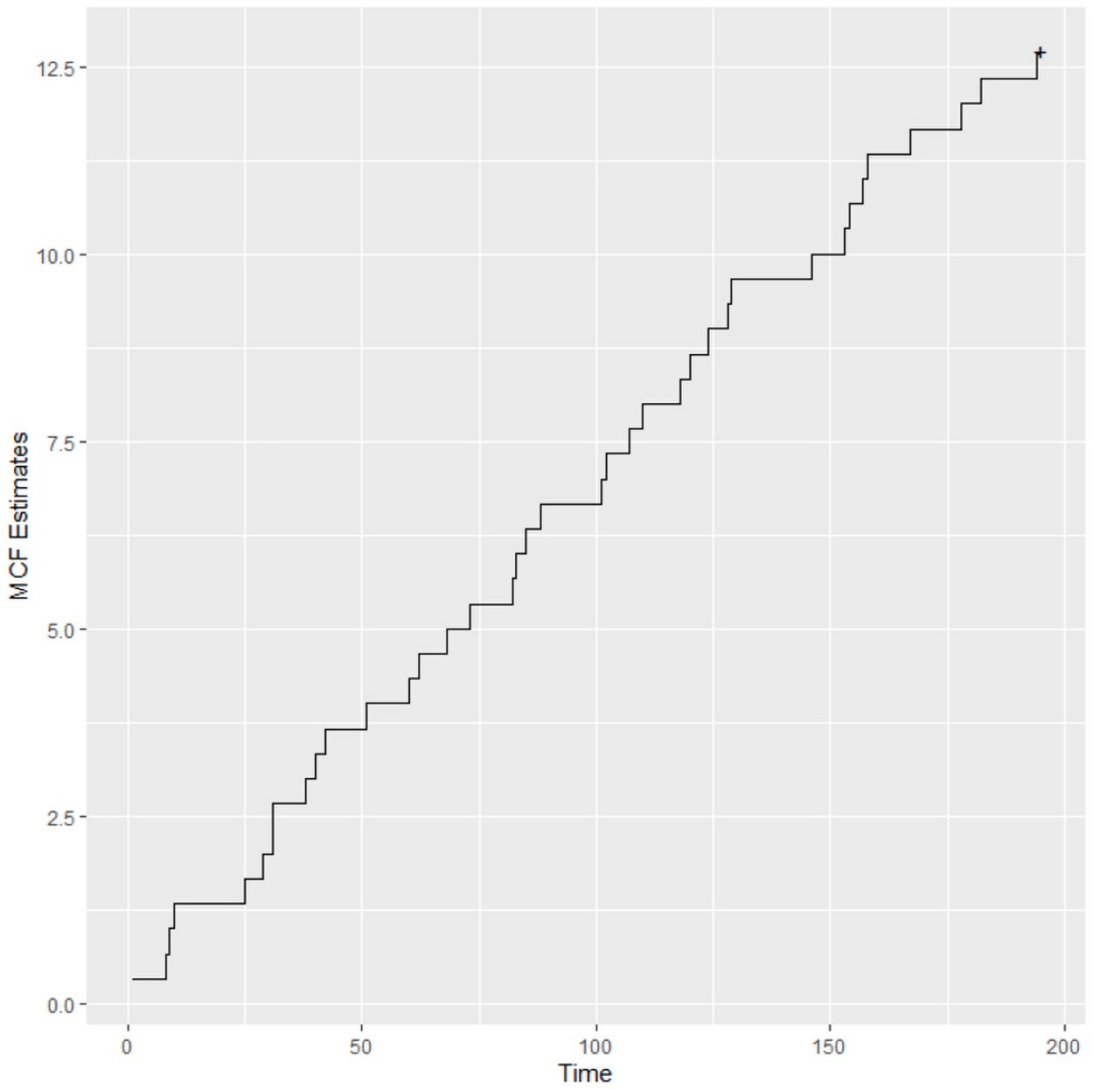
Mean cumulative function (MCF) estimate for data.

Fig 5 exhibits the mean cumulative function, i.e., the non-parametric Nelson-Aalen estimate for the Λ function. It is possible to observe that there is an indication that the intensity function is decreasing.

The adequacy of the PLP was verified through the Cramer von Mises goodness-of-fit test [41] for the sugarcane harvesters data set, whose hypothesis are given by

H_0_: PLP is adequate (data comes from the *F* distribution)
H_1_: PLP is not suitable (data does not come from the *F* distribution)

where *F* is the cumulative distribuction function of PLP given in (3) and the test statistic is calculated by

$$C_{N}^{2}=\frac{1}{12N}+\sum_{i=1}^{N}{\left[\frac{2i-1}{2N}-F(t_{i})\right]}^{2},$$ (22)

where $N=\sum_{i=1}^{k}n_{i}$ is the total number of observations time and *t*_1_ < … < *t_N_* are all the failure times. If the calculated $C_{N}^{2}$ value is greater than the tabulated value, then the null hypothesis that the data comes from the *F* distribution is rejected. To obtain the value of the *β* estimate, we calculate the maximum likelihood estimator of this parameter given in (9) and then we had $\hat{\beta }=0.899$ and considering the *N* = 38 failure times we obtain the value $C_{N}^{2}=0.023006$ of the test statistic. The respective p-value can be calculated using the *cvm.test* function from the *goftest* package [42] of the R software [38] from which we obtained a p-value equal to 0.9939 and thus we do not reject the null hypothesis and therefore the harvesters data set can be adjusted by a PLP.

Table 4 presents the model parameter estimates for the three methods presented in the study: MLE, Jeffreys posterior and reference posterior. Estimates were found using the estimators given by (9), (14) and (18). The confidence interval of the MLE was found through the asymptotic theory, that is, we find the variance of the estimates through the FIM and use the quantiles of the normal distribution. The Jeffreys and reference credibility intervals were found using the quantiles of their posterior distribution, given by (13) and (17), respectively. We can see that estimates are very close in all three methods used.

**Table 4:** Bayesian estimates for the parameters of the model.

Method	Parameter	Estimate	CI
MLE	$\hat{\beta }$	0.899	(0.614; 1.186)
	$\hat{\delta }$	12.667	(8.639; 16.694)
Jeffreys	$\hat{\beta }$	0.876	(0.637; 1.208)
	$\hat{\delta }$	12.667	(8.964; 16.999)
Reference	$\hat{\beta }$	0.876	(0.637; 1.208)
	$\hat{\delta }$	12.5	(9.104; 17.193)

The estimates suggest that the reliability of the systems is increasing over the time, that is, the intensity function is decreasing which is in accordance with the MCF plot. Moreover, it is expected that there are 12.5 failures in each sugarcane harvester in the period of 195 days.

The confidence intervals obtained through the MLE using the the asymptotic theory were (0.614; 1.186) for *β* and (8.639; 16.694) for *δ*. Hence, as expected, we observe that there is a significant difference in relation to those obtained through the reference posterior. This envisages that MLEs tend not to return reliable estimates for small samples. Finally, as we proved theoretically and from a simulation study, the Bayes estimates obtained with the overall reference posterior and given in Table 4 should be used.

## 6 Discussion

In this study, we have focused on the analysis of the reliability of multiple systems that can have multiple failures over time. Under the assumption of minimal repair, it was assumed that the failure has a power law intensity and, in order to facilitate the interpretation and the estimation process, we considered a useful reparameterization to obtain the Bayesian estimators.

The parameter estimators of the PLP model were obtained in closed-form expressions using the Bayesian approach. We discussed two objective priors known as Jeffreys prior and reference prior. The resulting posterior distributions lead to unbiased estimators. Considering the Jeffreys prior we proved that the resulting estimators lead to unbiased estimates for both parameters. On the other hand, the overall reference prior provided marginal posterior intervals with accurate frequentist coverage for both parameters, i.e., the prior is a matching prior. The obtained posterior distributions are proper and have one-to-one invariance property. An extensive simulation study was presented confirming our theoretical results. Overall, the Bayesian estimators with the Jeffreys prior returned improved estimates for both parameters as well as good credibility intervals and should be used to obtain the posterior estimates for the parameters. Note that, if the analysis is more interested in interval estimates, the posterior distribution with the reference prior should be used.

A real data set related to the breaking of the Chopper blade of sugarcane harvester was used to confirm the applicability of the proposed methodology. From the estimates obtained we can conclude that the blades over time have their reliability increased and during the 195 days of the study they have 12.5 failures as mean.

It is important to notice that this paper was formulated from our application of the harvesters. That is, we considered identical systems and truncation by time, because the harvesters come from the same company and were built under the same conditions. Also, they are subjected to the same working conditions, which leads us to consider them as identical systems. Also, time truncation was chosen due to the condition in which the harvesters are working (they stop working in a fixed period of time per year, which is exactly the harvest time). As a future work, we propose to consider situations where the systems are different and other kinds of truncation. Note that, in a new model, the parameterization used in this paper is not adequate, being necessary to use other parameterizations, such as the parametric form presented by Crow [28], and so, the new model will probably not have closed forms for the parameters estimators.
